# Impact of treatment delay due to the pandemic of COVID-19 on the efficacy of immunotherapy in head and neck cancer patients

**DOI:** 10.1186/s13045-020-01019-5

**Published:** 2020-12-11

**Authors:** Gaili Chen, Qiuji Wu, Huangang Jiang, Zheng Li, Xinying Hua, Xiaoyan Hu, Haijun Yu, Conghua Xie, Yahua Zhong

**Affiliations:** Department of Radiation and Medical Oncology, Hubei Key Laboratory of Tumor Biological Behaviors, Hubei Cancer Clinical Study Center, Zhongnan Hospital of Wuhan University, Wuhan, China

**Keywords:** Head and neck cancers, Immune checkpoint inhibitors, Treatment delay, Tumor response

## Abstract

Immunotherapy has been a new standard for recurrent/metastatic head and neck cancers (R/M HNC). One of the prominent characteristics of cancer immunotherapy is the induction of immune memory followed by endured treatment response. However, whether and how a treatment delay would impact on the efficacy of immunotherapy has not been well determined. During the outbreak of COVID-19, a number of cancer patients in Wuhan, the epicenter of the pandemic in China, had experienced long-lasting city lockdown and delay of immunotherapies. Here, we retrospectively analyzed 24 HNC patients treated with immune checkpoint inhibitors in our cancer institute prior to the outbreak of COVID-19 who were re-evaluated after the restoration of regular medical care. Of these 24 patients, 10 patients had achieved complete response (CR) or partial response (PR), 12 patients had achieved stable disease (SD), and 2 patients had received just one cycle treatment without efficacy evaluation before treatment delay. The median delay was 3.75 months (range 1.73–8.17 months). Re-evaluation after treatment delay revealed that ten patients (10/10) who achieved CR or PR, two patients (2/2) who received just one cycle treatment without efficacy evaluation and seven patients (7/12) who achieved SD before outbreak of COVID-19 maintained tumor response after treatment delay. Among the rest five patients who had achieved SD, four patients were re-evaluated as progressive disease (PD) due to treatment delay and one patient died after treatment interruption without re-evaluation. Our results from a small cohort of R/M HNC patients showed that treatment delay of three to four months might have mild, if any, impact on the efficacy of immunotherapy for patients with controlled disease.

## To the editor

Head and neck cancers are the ninth most common malignancy in the world [1]. Most patients present with locally advanced disease with a high risk of recurrence and metastasis [2]. Recent rapid progression in cancer immunotherapies has demonstrated unprecedented benefits of recurrent/metastatic head and neck cancers (R/M HNC) from immune checkpoint inhibitors (ICIs). As a result, ICIs alone in PD-L1 highly expressing settings or in combination with chemotherapies in the overall population has been recommended as new standard for R/M HNC [3–5].

One of the most distinguished characteristics of immunotherapies is the induction of caner-specific immunity and immune memory response, which could yield endured tumor responses observed in numerous clinical trials [3, 6, 7]. However, the interaction between cancer cells and their immune microenvironment is much complex and the mechanisms of cancer immunotherapy have not been fully understood. Treatment delay caused by reversible treatment toxicities, patient economic difficulties and other reasons is frequent, but its impact on treatment efficacy has not been well demonstrated. Cancer patients in Wuhan, the epicenter of the COVID-19 in China, provided valuable clues since they had experienced long-lasting city lockdown and a passive delay of treatments. Therefore, we analyzed the impact of treatment delay on HNC patients treated with immunotherapies in our cancer institute prior to the outbreak of COVID-19.

Twenty-four eligible HNC patients were identified (Table 1), including 19 males and 5 females with a median age of 58 years old. In total, 50% (12/24) of patients were diagnosed with oral cancer patients, 29.2% (7/24) with nasopharyngeal carcinoma and the other 20.8% (5/24) with hypopharyngeal carcinoma, sino-nasal malignant tumors, mandible carcinoma and parotid carcinoma. In total, 41.7% (10/24) of patients had metastatic diseases and 58.3% (14/24) had recurrent diseases. Immunotherapy-based therapy was administrated as first-line treatment in 87.5% (21/24), second-line in 4.2% (1/24) and fourth line in 8.3% (2/24) of patients. For ICIs, camrelizumab, toripalimab and pembrolizumab were used in 54.2% (13/24), 29.1% (7/24) and 16.7% (4/24) of patients, respectively. Nab-paclitaxel and gemcitabine were main chemotherapy agents used as combination therapy (22/24, 91.7%). The median time of treatment delay was 3.75 months (range 1.73–8.17 months). The last follow-up time was September 30, 2020. Tumor responses of each patient before treatment discontinuation and after treatment re-initiation were made by three oncologists independently according to the Response Evaluation Criteria in Solid Tumors (RECIST1.1) [8].

**Table 1:** Patients information

Patient ID	Gender	Age (years)	Primary diagnosis	Failure of disease	Treatment lines	Immunotherapy	Combined chemotherapy	Delay (months)
1	M	77	Mandible carcinoma	Recurrence	First line	Toripalimab	Nab-paclitaxel → GEM	3.27
2	M	73	Sino-nasal malignant tumors	Metastasis	First line	Pembrolizumab	Nab-paclitaxel	3.23
3	F	63	Oral cavity cancer	Metastasis	First line	Pembrolizumab	Nab-paclitaxel	3.50
4	M	51	Hypopharyngeal carcinoma	Recurrence	First line	Toripalimab	Nab-paclitaxel	3.23
5	M	56	Nasopharyngeal carcinoma	Recurrence	Second line	Toripalimab	S1	8.17
6	F	44	Nasopharyngeal carcinoma	Metastasis	First line	Camrelizumab	GEM	3.27
7	M	26	Parotid carcinoma	Metastasis	First line	Camrelizumab	Nab-paclitaxel	3.23
8	M	53	Oral cavity cancer	Recurrence	First line	Camrelizumab	GP → nab-paclitaxel	2.97
9	M	67	Oral cavity cancer	Recurrence	First line	Toripalimab	GEM	1.73
10	F	55	Oral cavity cancer	Recurrence	First line	Pembrolizumab	Nab-paclitaxel	4.57
11	M	50	Nasopharyngeal carcinoma	Metastasis	First line	Camrelizumab	Nab-paclitaxel → GEM	5.57
12	M	65	Nasopharyngeal carcinoma	Metastasis	First line	Camrelizumab	GEM	4.13
13	M	46	Nasopharyngeal carcinoma	Recurrence	First line	Camrelizumab	GP	3.50
14	M	30	Oral cavity cancer	Recurrence	First line	Camrelizumab	GP	4.13
15	M	58	Oral cavity cancer	Metastasis	First line	Camrelizumab	Nab-paclitaxel	3.13
16	M	69	Oral cavity cancer	Recurrence	First line	Pembrolizumab	Nab-paclitaxel	4.57
17	M	68	Oral cavity cancer	Recurrence	First line	Camrelizumab	Nab-paclitaxel	4.00
18	M	67	Hypopharyngeal carcinoma	Metastasis	Fourth line	Toripalimab	Vinorelbine	4.27
19	M	73	Nasopharyngeal carcinoma	Recurrence	First line	Camrelizumab	GEM	5.37
20	M	52	Oral cavity cancer	Recurrence	First line	Camrelizumab	GP → nab-paclitaxel	2.97
21	F	69	Oral cavity cancer	Metastasis	First line	Toripalimab	GEM	4.63
22	F	70	Oral cavity cancer	Metastasis	First line	Camrelizumab	GEM → nab-paclitaxel	4.37
23	M	58	Oral cavity cancer	Recurrence	First line	Camrelizumab	Nab-paclitaxel	3.13
24	M	49	Nasopharyngeal carcinoma	Recurrence	Fourth line	Toripalimab	Nab-paclitaxel	6.13

M, male; F, female; GEM, gemcitabine; GP, gemcitabine plus cisplatin; S1, gimeracil and oteracil potassium capsules

**Fig. 1:**
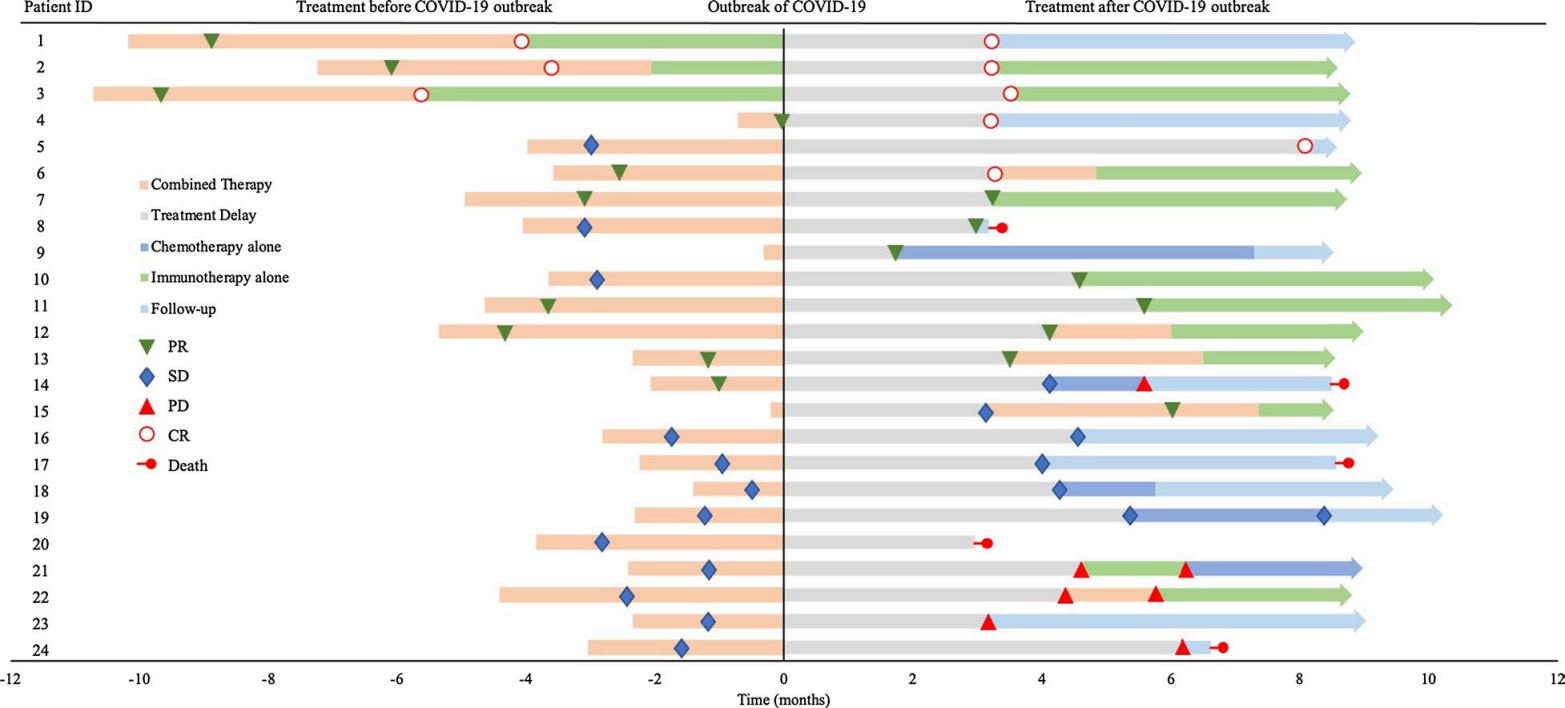
Impact of treatment delay on the efficacy of immunotherapy in head and neck cancer patients. *CR* complete response, *PR* partial response, *PD* progressive disease, *SD* stable disease

Of the 24 patients enrolled, ten patients had achieved CR or PR, two patients had received just one cycle treatment without efficacy evaluation, and twelve patients had achieved SD before outbreak of COVID-19. For the ten patients who had achieved CR or PR before treatment interruption, no disease progression was observed upon re-evaluation after treatment delay. Interestingly, disease control was also seen in those two patients who had received just only one cycle treatment without efficacy evaluation and in seven out of twelve patients who had only achieved SD before treatment interruption (Fig. 1). On the other hand, four patients (4/12) with SD were re-evaluated as PD due to treatment delay and the other one patient (1/12) died after treatment interruption without re-evaluation (Fig. 1). Importantly, we noted that most patients experiencing treatment response and maintained clinical benefit (CR and PR) were those who had longer prior treatment exposure (median exposure time: 4.03 months vs 2.33 months). This might imply that treatment interruption in patients who had only received short-term immunotherapy should be discouraged.

Five patients died until the last follow-up. Patient 8 died of an accidental asphyxia even though he had PR disease. After 4.13 months treatment delay, patient 14 was evaluated as SD with enlarged lesion, he gave up immunotherapy and received only chemotherapy, and eventually he died of disease progression. Patient 17 was evaluated as SD after treatment delay, but he refused further treatment and died 8 months after the treatment interruption. Patient 20 died due to treatment interruption, with enlarged SD status before COVID-19 outbreak. Patient 24 died of mucosal ulcer bleeding as a result of disease progression. In all, no ≥ Grade 3 immune-related toxicity was observed in all 24 patients.

Collectively, these observations suggested that for those who had achieved CR/PR response to immunotherapy and who had a longer prior treatment exposure, a relatively short treatment delay of about three to four months did not lead to significant treatment failure. Yet for those who had only stable diseases, it is important to find alternative treatments during the treatment interruption since they are more likely to experience disease progression. Importantly, re-initiation of immunotherapy in these patients did not reverse disease progression.

As this is a retrospective study and the sample size is relatively small, these conclusions need to be interpreted with caution.

## Data Availability

All data generated or analyzed during this study are included in this published article.

## Abbreviations

CR: Complete response
ICIs: Immune checkpoint inhibitors
OS: Overall survival
PD: Progressive disease
PR: Partial response
RECIST: Response Evaluation Criteria in Solid Tumors
R/M HNC: Recurrent/metastatic head and neck cancers
SD: Stable disease
